# *Helicobacter pylori* VacA Targets Myeloid Cells in the Gastric Lamina Propria To Promote Peripherally Induced Regulatory T-Cell Differentiation and Persistent Infection

**DOI:** 10.1128/mBio.00261-19

**Published:** 2019-03-19

**Authors:** Aleksandra Altobelli, Michael Bauer, Karelia Velez, Timothy L. Cover, Anne Müller

**Affiliations:** a Institute of Molecular Cancer Research, Zurich, Switzerland; b Vanderbilt University Medical Center and Veterans Affairs Tennessee Valley Healthcare System, Nashville, Tennessee, USA; New York University School of Medicine

**Keywords:** T-cell immunity, T cells, dendritic cells, host-cell interactions, immunomodulation, mucosal infection, regulatory T cells, macrophages

## Abstract

The gastric bacterium Helicobacter pylori causes a persistent infection that is directly responsible for gastric ulcers and gastric cancer in some patients and protective against allergic and other immunological disorders in others. The two outcomes of the *Helicobacter*-host interaction can be modeled in mice that are infected as immunocompetent adults and as neonates, respectively. Here, we have investigated the contribution of the *Helicobacter* immunomodulator VacA to H. pylori-specific local and systemic immune responses in both models. We found that neonatally infected mice are colonized at higher levels than mice infected as adults and fail to generate effector T-cell responses to the bacteria; rather, T-cell responses in neonatally infected mice are skewed toward Foxp3-positive (Foxp3^+^) regulatory T cells that are neuropilin negative and express RORγt. We found these peripherally induced regulatory T cells (pTregs) to be enriched, in a VacA-dependent manner, not only in the gastric mucosa but also in the lungs of infected mice. Pulmonary pTreg accumulation was observed in mice that have been infected neonatally with wild-type H. pylori but not in mice that have been infected as adults or mice infected with a VacA null mutant. Finally, we traced VacA to gastric lamina propria myeloid cells and show that it suppressed interleukin-23 (IL-23) expression by dendritic cells and induced IL-10 and TGF-β expression in macrophages. Taken together, the results are consistent with the idea that H. pylori creates a tolerogenic environment through its immunomodulator VacA, which skews T-cell responses toward Tregs, favors H. pylori persistence, and affects immunity at distant sites.

## Abstract (precis)

Helicobacter pylori has coexisted with humans for at least 60.000 years and has evolved persistence strategies that allow it to evade host immunity and colonize its host for life. The VacA protein is expressed by all H. pylori strains and is required for high-level persistent infection in experimental mouse models. Here, we show that VacA targets myeloid cells in the gastric mucosa to create a tolerogenic environment that facilitates regulatory T-cell differentiation, while suppressing effector T-cell priming and functionality. Tregs that are induced in the periphery during H. pylori infection can be found not only in the stomach but also in the lungs of infected mice, where they are likely to affect immune responses to allergens.

## INTRODUCTION

Infection with the gastric bacterium Helicobacter pylori is associated with a range of gastric disorders that include peptic ulcers and gastric cancer but is now also known to have systemic consequences for the host that manifest at distant sites. For example, infected children exhibit reduced growth rates and suffer more commonly from anemia than their uninfected counterparts and, on the other hand, show reduced susceptibility to atopy and allergic conditions in general (1–3). *Helicobacter* eradication by combination therapy with two to three antibiotics is a viable and cost-efficient strategy to reduce gastric cancer risk; however, not all carriers of H. pylori benefit equally from the successful eradication of H. pylori (4, 5). In particular, subgroup analyses of adults presenting with preneoplastic lesions at the time of eradication therapy show differential rates of treatment success. Only patients with atrophic or nonatrophic gastritis, and not patients that have progressed to metaplasia or dysplasia, appear to benefit from eradication therapy through reduced gastric cancer risk (5), although this notion was recently challenged by a study showing that H. pylori eradication may still reduce the risk of development of metachronous gastric cancer in patients who have already undergone endoscopy for early gastric cancer (6). As infected children are much less likely to develop H. pylori-related symptoms than infected adults (7), routine eradication therapy (“test and treat”) is not recommended in this age group (8). Furthermore, children are more likely to benefit from harboring H. pylori, and eradication of the infection in children is projected to incur a significant cost. The identification of biomarkers that allow accurate predictions of who will benefit from eradication therapy and who will not, and of who will progress along the path to gastric cancer and who will not, therefore is widely considered to be of high priority. Attempts have been made to stratify patients based on the frequencies in blood of regulatory T cells (Tregs), especially of the subset of Tregs expressing the anti-inflammatory cytokine interleukin-10 (IL-10) (9). That work showed that high frequencies of IL-10-positive (IL-10^+^) Tregs in the circulation of H. pylori carriers were inversely associated with allergen-specific IgE concentrations and that blocking of IL-10 *in vitro* restored IgE responses (9). An earlier study had already pointed to IL-10^+^ Treg responses in the stomach as being predictive of asymptomatic carriage of H. pylori; in contrast, low Treg counts and high frequencies of Th1 and Th2 responses were associated with peptic ulcers (10). Such protective Treg responses are particularly characteristic of infected children, who generally present with fewer symptoms, lower levels of histologically evident pathology, and more pronounced Treg responses than adult counterparts infected with similarly aggressive H. pylori strains (7). High numbers of gastric IL-10^+^ Tregs in children have been linked to reduced Th17 responses to the infection, which may well account for the reduced immunopathology in that age group (11). The immunophenotypic data from infected children and adults align well with observations from epidemiological studies on asthma and allergy risk in relation to H. pylori infection. In this context, H. pylori confers a stronger protective effect in children than in adults, and early-onset asthma shows a particularly strong inverse correlation with H. pylori infection (12, 13). The stronger inverse association of asthma with H. pylori in children than in adults was confirmed in several meta-analyses (14).

Previous work in our laboratory provided experimental evidence from mouse models that link early-life H. pylori exposure to sustained protection against ovalbumin- or house dust mite (HDM)-induced allergic airway inflammation (15–17), dextran sulfate sodium (DSS)-induced colitis (18), and peanut- or ovalbumin-induced food allergy (19). The protective effects are either not seen at all or are much weaker in mice infected as adults (15). We have further reported that early-life exposure to H. pylori induces immune tolerance, driven by Foxp3^+^ Tregs, that prevents immunopathology and excessive effector T-cell responses to the infection and permits high-level (asymptomatic) colonization (20). The neonatal infection model thus phenocopies several aspects of the host/H. pylori interaction in children and asymptomatic carriers (7, 10, 11) and lends itself to mechanistic studies and biomarker discovery. We and others have used the neonatal and adult infection models to identify both host and H. pylori determinants that promote immune tolerance; Treg induction; and high-level, persistent colonization. The H. pylori immunomodulators gamma-glutamyl-transpeptidase (gGT) and vacuolating cytotoxin (VacA) have emerged as critical factors shaping the outcome of H. pylori infection. Interestingly, both proteins are secreted and act on T cells as well as myeloid cells to prevent T-cell activation and function on the one hand (21, 22) and to induce tolerogenic activities in dendritic cells (DCs) on the other (23, 24). Infection of mice with H. pylori strains deficient in either of the two immunomodulators is associated with improved immune control and clearance (24–26) and with a reduced capability of DCs to prime Treg differentiation in the periphery, relative to infection of mice with wild-type (WT) H. pylori strains. Both gGT and VacA can be used in purified or recombinant form to reduce the severity of allergy symptoms in prophylactic settings, provided that the proteins are administered early in life (27).

Here, we set out to identify immune correlates of, and to gain mechanistic insights into, the differential responses of neonatal and adult mice to H. pylori strains that either express the VacA protein or not. We use tracking strategies and multicolor flow cytometry of leukocyte populations in the gastric mucosa and the lung (a distant site at which protective effects of gastric H. pylori infection are detected) to quantify differential myeloid and T-cell responses as a function of age at first exposure and of VacA expression. We show here that neonatal and adult infected mice differ in their predominant T-cell responses to the infection and that VacA acts directly on macrophages and DCs to promote the differentiation and maintenance of Tregs but not of Th17 cells; the effects of gastric H. pylori colonization are further evident in the lungs of neonatal but not adult infected mice even prior to allergen exposure and shape allergen-specific immune responses at this distant site.

## RESULTS

### VacA promotes immune tolerance by skewing gastric T-cell responses in favor of peripherally induced RORγt^+^ regulatory T cells.

**FIG 1:**
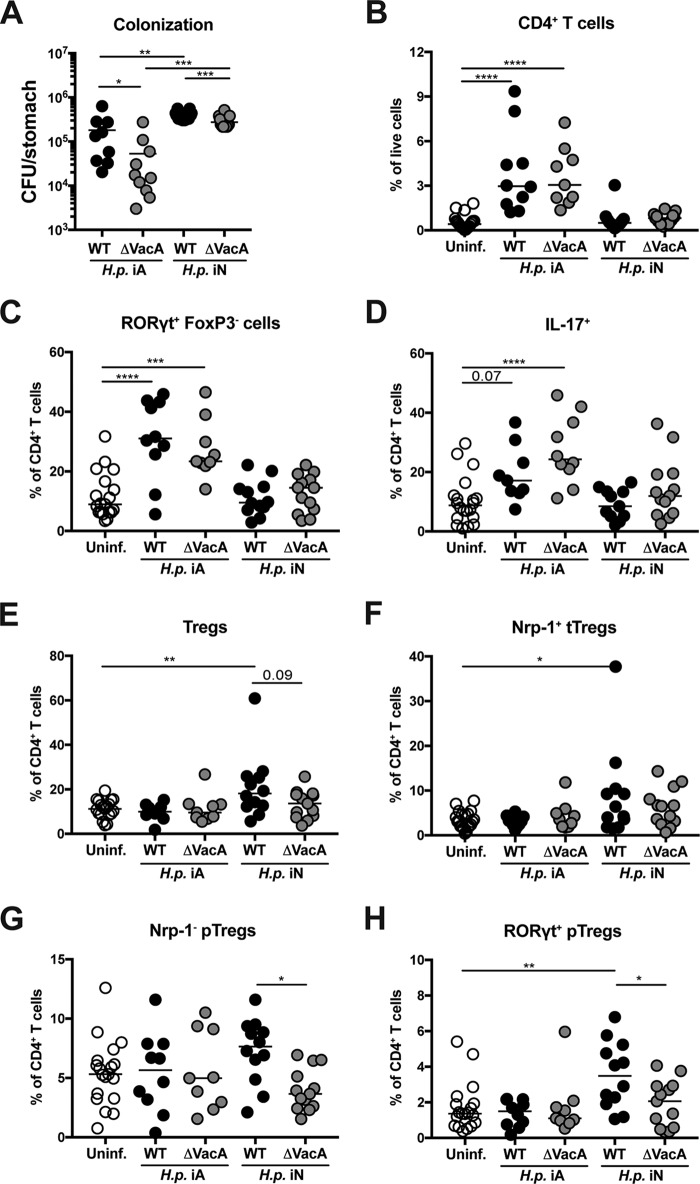
Gastric T-cell responses in mice infected neonatally with VacA-proficient H. pylori are skewed in favor of peripherally induced RORγt^+^ regulatory T cells. C57BL/6 mice were infected at either 7 days of age (i.e., were infected neonatally [iN]) or 6 weeks of age (i.e., were infected as adults [iA]) with H. pylori PMSS1 or its isogenic VacA-deficient mutant (ΔVacA), and colonization levels and gastric lamina propria (LP) T-cell responses were analyzed by FACS 6 weeks later. (A) H. pylori (*H.p.*) colonization, as assessed by plating and colony counting. (B) Frequencies of CD4^+^ T cells among all live LP cells of the mice as indicated in panel A along with uninfected controls. (C) Frequencies of RORγt^+^ Foxp3^−^ cells among all CD4^+^ T cells. (D) Frequencies of IL-17^+^ CD4^+^ T cells, as assessed by restimulation with PMA/ionomycin, followed by intracellular cytokine staining. (E) Frequencies of Foxp3^+^ regulatory T cells (Tregs) among all CD4^+^ T cells. (F) Frequencies of neuropilin-1-positive (Nrp-1^+^) Foxp3^+^ thymus-derived Tregs among all CD4^+^ T cells. (G) Frequencies of Nrp-1^−^ Foxp3^+^ peripherally induced Tregs (pTregs) among all CD4^+^ T cells. (H) Frequencies of RORγt^+^ Nrp-1^−^ pTregs among all CD4^+^ T cells. Pooled data from two independent experiments are shown throughout, with *n* = 10 to 13 per condition. Horizontal lines indicate medians; statistical analyses were done using ANOVA with Dunn’s multiple-comparison correction. *, *P* < 0.05; **, *P* < 0.01; ***, *P* < 0.005; ****, *P* < 0.001. The gating strategy for Treg and effector T-cell populations is shown in Fig. S1.

We showed earlier that a VacA-expressing H. pylori strain, but not its isogenic null mutant, is capable of suppressing gastric H. pylori-specific effector T-cell responses and maintaining high-level persistent colonization (24). To investigate in more detail whether VacA proficiency and the age of the mice at the time of infection affect gastric T-cell responses, we conducted a multicolor flow cytometric analysis of gastric lamina propria (LP) T-cell populations of mice that had been infected at 7 days or 6 weeks of age (neonatal or adult infection, respectively) with wild-type H. pylori or its isogenic VacA-deficient mutant. Whereas neonatally infected mice were characterized by high-level colonization but no detectable CD4^+^ T-cell infiltration, their counterparts that had been infected as immunocompetent adults were colonized at lower levels and showed strong CD4^+^ T-cell recruitment (Fig. 1A and B; see also Fig. S1A in the supplemental material). VacA-deficient bacteria colonized mice at significantly lower levels than the parental strain in both the neonatal and the adult infection model but showed similar levels of CD4^+^ T-cell infiltration overall (Fig. 1A and B). Staining for intracellular IL-17 and for the transcription factor RORγt revealed robust Th17-polarized effector T-cell responses in the adult infection scenario that were largely absent in neonatally infected mice (Fig. 1C and D; see also Fig. S1B). In contrast, neonatally infected mice exhibited higher frequencies of Foxp3^+^ regulatory T cells (Tregs), a result that was at least partly dependent on VacA (Fig. 1E). Both peripherally induced, neuropilin-negative pTregs and thymus-derived, neuropilin-positive tTregs were somewhat overrepresented in neonatally infected mice (Fig. 1F and G). The differences in pTreg frequencies between adult and neonatally infected mice on the one hand and between wild-type (WT) and VacA mutant-infected mice on the other were largely attributable to the RORγt-positive pTreg subset (Fig. 1H), which represents the dominant pTreg subset in the gastric LP after H. pylori infection (see schematic in Fig. S1A). The results indicate that VacA expression by the bacteria and the age at the time of infection both have a strong impact on T-cell recruitment and polarization as well as on infection control.

10.1128/mBio.00261-19.1 FIG S1 Gating strategy for the identification and quantification of various T-cell populations in the gastric lamina propria. (A) Intracellular staining of Foxp3, RORγt, and Tbet in combination with surface staining of CD3, CD4, and neuropilin allows the differentiation of various effector T-cell and regulatory T-cell populations. (B) Restimulation with PMA/ionomycin, followed by intracellular staining for IL-17 expression, allows the quantification of cytokine-expressing Th17 cells. Download FIG S1, TIF file, 4.4 MB. Copyright © 2019 Altobelli et al. 2019 Altobelli et al. This content is distributed under the terms of the Creative Commons Attribution 4.0 International license.

### VacA targets macrophages and dendritic cells in the gastric lamina propria.

**FIG 2:**
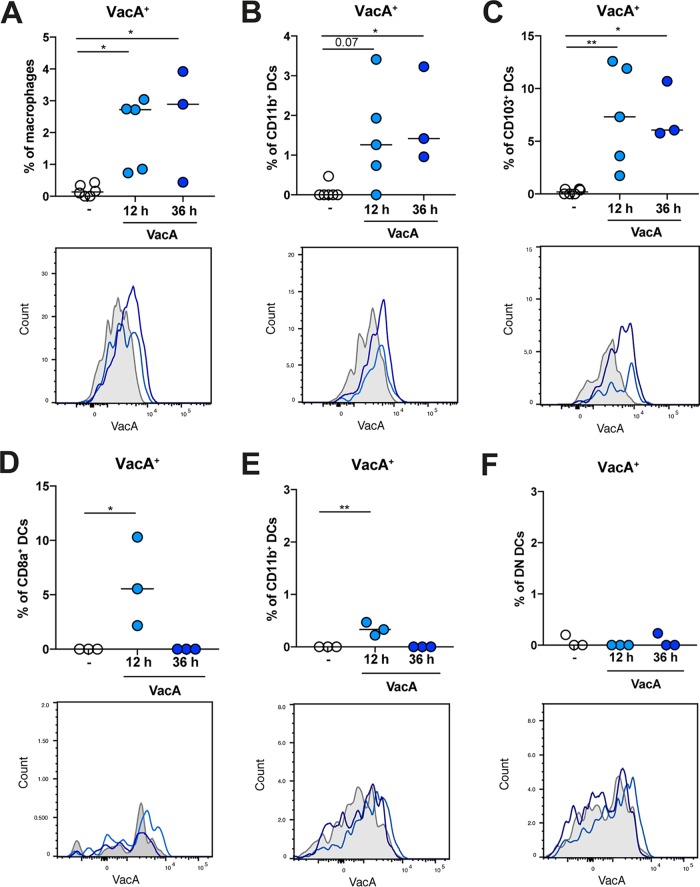
VacA associates with myeloid cells in the gastric lamina propria and the Peyer’s patches. Mice received a single dose of 20 μg of purified VacA by oral gavage and were sacrificed 12 or 36 h later. Gastric LP (A to C) and Peyer’s patch (D to F) single-cell preparations were subjected to multicolor flow cytometry with antibodies allowing the identification of various myeloid cell populations as well as an antiserum specific for VacA. (A to C) Frequencies of VacA-positive cells among F4/80^+^ Ly6C^−^ Ly6G^−^ MHCII^+^ macrophages (A), among CD11b^+^ CD103^−^ F4/80^−^ Ly6C^−^ Ly6G^−^ MHCII^+^ DCs (B), and among CD103^+^ CD11b^−^ F4/80^−^ Ly6C^−^ Ly6G^−^ MHCII^+^ DCs (C) in the gastric LP. Frequencies are shown along with representative histograms. (D to F) Frequencies of VacA-positive cells among CD8a^+^CD11b^−^ MHCII^+^ CD11c^+^ DCs (D), among CD11b^+^CD8a^−^ MHCII^+^ CD11c^+^ DCs (E), and among CD8a^−^ CD11b^−^ MHCII^+^ CD11c^+^ double-negative (DN) DCs (F) in the Peyer’s patches. Frequencies are shown along with representative histograms. Results presented in panels A to F are representative of three independently conducted experiments. Horizontal lines indicate medians; statistical analyses were done using ANOVA with Dunn’s multiple-comparison correction. The gating strategy for myeloid populations is shown in Fig. S2.

To address how, and through which cell types, VacA contributes to T-cell skewing and immunomodulation, we first assessed which leukocyte populations are targeted by VacA in the gastric LP. Our attempts at tracking VacA secreted by live bacteria were futile, as the only VacA-specific antiserum we have at our disposal that detects the native protein was raised against the s1m1 variant of VacA (28); our mouse-colonizing H. pylori PMSS1 strain expresses the s2m2 VacA variant that is not well detected by the serum. Therefore, we resorted to a strategy that involved oral gavage of mice with purified H. pylori s1m1 VacA, followed by the flow cytometric analysis of VacA signals in various leukocyte populations of the gastric LP at 12 and 36 h postadministration. VacA could be traced to three myeloid populations, i.e., F4/80-positive macrophages, CD11b^+^ DCs, and CD103^+^ DCs (see Fig. S2A for the gating strategy used for the identification of these populations), at both 12 and 36 h postadministration (Fig. 2A to C), but the signals faded thereafter (data not shown). VacA-positive cells were relatively rare among all macrophage and DC populations, with frequencies generally under 10%, but signals were clearly discernible and not observed in untreated mice (Fig. 2A to C). To examine whether VacA is also sampled by antigen-presenting cells in the lower gastrointestinal (GI) tract, we harvested Peyer’s patches and analyzed them for possible VacA signals. CD8α^+^ and, to a lesser extent, CD11b^+^ DCs, but no other DC, and no other myeloid populations of that lymphoid tissue, showed evidence of VacA positivity at 12 h postadministration (Fig. 2D to F); fluorescently labeled ovalbumin, which served as a model antigen and positive control, showed similar kinetics and distributions in Peyer’s patches (Fig. S2B).

10.1128/mBio.00261-19.2 FIG S2 VacA targets various myeloid populations in the gastric LP and peritoneum, depending on the route of delivery. (A) Gating strategy for the identification of CD103^+^ and CD103^+^ CD11b^+^ DCs among all live CD45^+^ CD11c^+^ MHCII^hi^ F4/80^−^ leukocytes and for the identification of CD11b^+^ DCs and of macrophages among live CD103^−^ MHCII^+^ Ly6G^−^ Ly6C^−^ leukocytes. (B) OVA-AF647 fluorescence of CD11b^+^ DCs in Peyer’s patches of mice orally gavaged with 20 μg ovalbumin and analyzed 12 and 36 h later. (C) Frequencies of VacA-positive large peritoneal macrophages (LPMs) and small peritoneal macrophages (SPMs) isolated from the peritoneum of mice that received 20 μg purified VacA intraperitoneally and were analyzed 2, 6, and 16 h later. Summary plots and representative overlaid histograms are shown in panels B and C. (D and E) Expression of the indicated transcripts, as assessed by qRT-PCR, of LPMs (D) and SPMs (E) isolated by peritoneal lavage from mice that had received 20 μg of either purified WT VacA or mutant VacA by i.p. injection 6 h earlier. Data from one experiment representative of two are shown in panels B to E. Statistical analyses were done using ANOVA with Dunn’s multiple-comparison correction throughout. Download FIG S2, TIF file, 2.6 MB. Copyright © 2019 Altobelli et al. 2019 Altobelli et al. This content is distributed under the terms of the Creative Commons Attribution 4.0 International license.

VacA has immunomodulatory activities not only when it is delivered in the context of a live infection but also when administered orally or intraperitoneally (i.p.) to pups early in life (19, 27). To mimic this scenario and monitor the fate of VacA in the peritoneum over time, we subjected mice to i.p. injection once with 20 μg of purified VacA, isolated peritoneal macrophages 2, 6, and 16 h later by peritoneal lavage, and subjected the cells to VacA staining. Two predominant, developmentally distinct subsets of macrophages (termed “small” and “large” peritoneal macrophages) (29) were retrieved using this procedure and were found to be strongly positive for VacA at 2 and 6 h postadministration, with signals fading at 16 h postdelivery (Fig. S2C). To study the transcriptional signature of macrophages that have been exposed to VacA *in vivo*, we isolated large and small peritoneal macrophages from mice that had been treated for 6 h with either wild-type VacA protein or an inactive VacAΔ6–27 mutant protein (which is defective in membrane channel formation due to absence of a hydrophobic domain near the N terminus of the protein) (30, 31). Wild-type VacA, but not the mutant, induced the expression of several examined genes in both large and small macrophages, including those encoding the cytokines tumor necrosis factor alpha (TNF-α), IL-10, and IL-6, as well as the enzyme cyclooxygenase 2 (COX2; encoded by the *PTGS2* gene) (Fig. S2D and E). The response of macrophages was consistent with a mixed response consisting of both pro- and anti-inflammatory mediators. The combined data suggest that VacA targets myeloid cells in its natural environment, i.e., the gastric mucosa and Peyer’s patches, and also targets peritoneal macrophages in an interventional setting designed to promote immune tolerance.

### VacA suppresses IL-23 expression by gastric CD11b^+^ DCs and induces IL-10 and TGF-β expression by macrophages.

**FIG 3:**
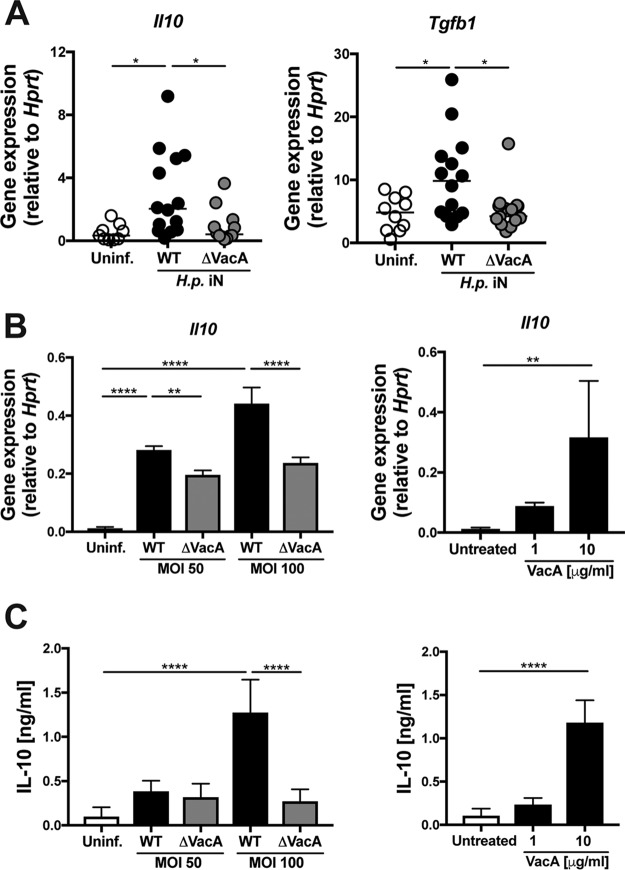
VacA activates IL-10 and TGF-β expression by macrophages in the gastric LP. (A) CX_3_CR1^+/GFP^ mice were infected at 7 days of age (iN) with H. pylori PMSS1 or its isogenic VacA-deficient mutant (ΔVacA) and sacrificed 6 weeks later. F4/80^+^ Ly6C^lo^ MHCII^+^ CX3CR1^hi^ macrophages were sorted from gastric LP preparations of individual mice and subjected to reverse transcriptase quantitative PCR (qRT-PCR) analyses of *Il10* (left) and *Tgfb1* (right). Results shown in panel A were pooled from two independent studies. Each symbol represents sorted cells from a single mouse. (B and C) Bone marrow-derived murine macrophages were infected with H. pylori PMSS1 or its ΔVacA mutant at the indicated MOIs, or treated with the indicated concentrations of purified VacA for 6 h. Cells were subjected to qRT-PCR analyses of *Il10* expression (B), and their supernatants were subjected to IL-10 quantification by ELISA (C). Data in panels B and C are representative of results from three independent studies. Means plus standard errors of the means (SEM) are shown; statistical analyses were done using ANOVA with Dunn’s multiple-comparison correction. *, *P* < 0.05; **, *P* < 0.01; ***, *P* < 0.005; ****, *P* < 0.001.

Naïve T cells differentiate into RORγt-positive Th17 cells if they are exposed to their corresponding cognate antigen on antigen-presenting cells in environments that are rich in IL-6 and TGF-β. A third Th17-promoting cytokine, IL-23, acts to maintain Th17 differentiation but is not required during the priming phase in lymph nodes. We have shown previously that H. pylori encounters monocytes and macrophages as well as CD11b^+^ DCs, but not CD103^+^ DCs, in the gastric LP as judged by tracking of an red fluorescent protein (RFP)-expressing H. pylori strain (32). All three populations are recruited to the H. pylori-infected gastric LP (32), and their levels of recruitment do not differ measurably if the infecting strain lacks VacA (data not shown). To assess whether VacA contributes to cytokine production by dendritic cells or macrophages, we subjected pure populations of F4/80^−^ CD11b^+^ DCs and F4/80^+^ Ly6c^lo^ CX_3_CR1^hi^ macrophages from LP preparations of mice that had been infected neonatally with WT or ΔVacA H. pylori to fluorescence-activated cell sorter (FACS) analysis and determined their levels of expression of cytokines relative to cells from uninfected controls. We found several typical DC cytokines, including IL-23, IL-12, IL-6, IL-10, and TGF-β, to be upregulated in DCs from infected mice; most of these were similarly highly expressed in DCs from WT and ΔVacA H. pylori*-*infected mice (Fig. S3A and B and data not shown). One notable exception was IL-23, which was expressed at significantly higher levels in DCs from ΔVacA H. pylori*-*infected stomachs than in DCs from WT H. pylori*-*infected stomachs (Fig. S3A and B). Macrophages also exhibited VacA-dependent responses to H. pylori: only macrophages sorted from LP preparations of mice that had been infected with WT but not with ΔVacA H. pylori expressed IL-10 and TGF-β, whereas the level of expression of IL-12 was unaffected by the VacA status (Fig. 3A; see also Fig. S3C). A similar dependence of IL-10 induction in macrophages on VacA was observed *in vitro* with bone marrow-derived macrophages infected with H. pylori, at both the transcript and protein levels (Fig. 3B and C). A second strain of H. pylori expressing s1m1 VacA showed the same VacA dependence regarding IL-10 induction (Fig. S3D). Furthermore, exposure to purified s1m1 VacA was sufficient to dose-dependently induce IL-10 expression and secretion in macrophages *in vitro* (Fig. 3B and C). The combined results suggest that VacA modulates the responses of DCs and macrophages to H. pylori, resulting in a milieu that promotes Treg over Th17 differentiation and/or expansion.

10.1128/mBio.00261-19.3 FIG S3 VacA suppresses IL-23 expression by DCs but has no effect on IL-12 expression by macrophages. (A and B) CD11b^+^ DCs that were sorted from LP preparations of mice that had been infected neonatally with either WT or ΔVacA H. pylori were subjected to RNA extraction and qRT-PCR using primers specific for *Il23a*, *Tgfb1*, and *Il10*. Results of a representative study are shown in panel A, where every data point represents cells sorted from one mouse. Median values from two (*Tgfb1*) or three (*Il10* and *Il23a*) independent experiments are shown in panel B. (C) Macrophages that were sorted from LP preparations of mice that had been infected neonatally with either WT or ΔVacA H. pylori were subjected to RNA extraction and qRT-PCR using primers specific for the IL-12 β-chain. Mice shown in panel C are the same as those shown in Fig. 3A. (D) Bone marrow-derived murine macrophages were infected with H. pylori G27 or its ΔVacA mutant at the indicated MOIs. Cells were subjected to qRT-PCR analyses of *Il10* expression. Data in panel D are representative of results from two independent experiments. Means plus SEM are shown; statistical analyses were done using ANOVA with Dunn’s multiple-comparison correction throughout. Download FIG S3, TIF file, 4.1 MB. Copyright © 2019 Altobelli et al. 2019 Altobelli et al. This content is distributed under the terms of the Creative Commons Attribution 4.0 International license.

### Gastric infection with VacA-proficient H. pylori skews lung T-cell populations toward Th1 and regulatory T cells.

**FIG 4:**
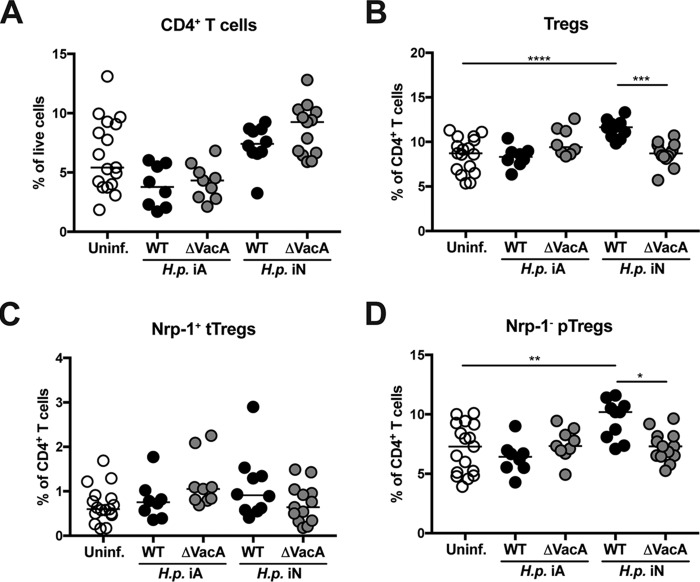
Neonatal H. pylori infection is associated with a VacA-dependent systemic Treg response that manifests in the lung. C57BL/6 mice were infected either at 7 days of age (i.e., were infected neonatally [iN]) or 6 weeks of age (i.e., were infected as adults [iA]) with H. pylori PMSS1 or its isogenic VacA-deficient mutant (ΔVacA), and pulmonary T-cell responses were analyzed by FACS 6 weeks later. (A) Frequencies of CD4^+^ T cells among all live cells. (B) Frequencies of Foxp3^+^ Tregs among all CD4^+^ T cells. (C) Frequencies of Nrp-1^+^ thymus-derived tTregs among all CD4^+^ T cells. (D) Frequencies of Nrp-1^−^ peripherally induced pTregs among all CD4^+^ T cells. Data pooled from two independent experiments are shown throughout, with *n* = 10 to 13 per condition. Horizontal lines indicate medians; statistical analyses were done using ANOVA with Dunn’s multiple-comparison correction. *, *P* < 0.05; **, *P* < 0.01; ***, *P* < 0.005; ****, *P* < 0.001. The gating strategy for pulmonary Tregs is the same as for gastric Tregs and is shown in Fig. S1.

H. pylori causes a strictly localized gastric infection and is not known to spread to other parts of the body. Nevertheless, it has strong modulatory properties that manifest in distant organs, especially the lung. VacA is one of the major immunomodulatory molecules that contribute to the systemic effects of H. pylori infection (19, 24, 27). In order to identify immunological correlates of the reduced airway inflammation that is a hallmark of H. pylori-infected mice (15–17), we profiled pulmonary T-cell populations of mice that had been infected as adults or as neonates with H. pylori. Whereas the overall frequencies of pulmonary CD4^+^ T cells were not measurably affected by gastric H. pylori infection irrespective of the age at the time of infection (Fig. 4A), we detected shifts in pulmonary Treg frequencies that were consistent with those observed in the gastric LP. Interestingly, we found that the lungs of mice that had been infected neonatally with wild-type H. pylori but not VacA mutant H. pylori exhibited increased frequencies of Foxp3^+^ Tregs, which, as in the case of the gastric LP, could be attributed to the peripherally induced neuropilin-negative pTreg subset (Fig. 4B to D). Frequencies of pulmonary neuropilin-positive tTregs were unchanged (Fig. 4C). Mice that had been infected as adults rather than during early life did not exhibit the described changes in pulmonary Treg cell populations (Fig. 4B) but rather showed pulmonary T-cell skewing toward Th1 and Th17 cells (Fig. S4). The combined results demonstrate that local infection of the gastric mucosa with H. pylori has systemic consequences that manifest in the lung in the appearance of a population of peripherally induced Tregs; this Treg population is seen only under conditions of neonatal, tolerogenic H. pylori infection and depends on the immunomodulator VacA.

10.1128/mBio.00261-19.4 FIG S4 Pulmonary T cells are skewed towards Th1 and Th17 cells during adult infection with H. pylori. Mice were infected at 6 weeks (iA) or 7 days (iN) of age with wild-type or ΔVacA H. pylori for 1 month, and their pulmonary T-cell compartment was analyzed by FACS relative to uninfected controls. The expression of intracellular IFN-γ and IL-17 was analyzed upon restimulation with PMA/ionomycin; the expression of RORγt and Foxp3 was analyzed in fixed, permeabilized cells. In panels A to C, horizontal lines indicate medians; statistical analyses were done using ANOVA with Dunn’s multiple-comparison correction. *, *P* < 0.05; **, *P* < 0.01; ***, *P* < 0.005; ****, *P* < 0.001. Data represent pooled results from two studies. Download FIG S4, TIF file, 1.3 MB. Copyright © 2019 Altobelli et al. 2019 Altobelli et al. This content is distributed under the terms of the Creative Commons Attribution 4.0 International license.

## DISCUSSION

The objectives of this work were to (i) gain a detailed understanding of the function of H. pylori VacA in immunity and immune tolerance to H. pylori and (ii) investigate the mechanistic basis underlying the differential responses of neonatal and adult mice to H. pylori. We have previously demonstrated in a model of H. pylori infection of adult, immunocompetent mice that VacA is required for high-level persistent colonization (24). Here, we have extended these findings to the neonatal infection model and show that VacA promotes colonization under both conditions. We have further used a flow cytometric approach to trace VacA in the murine gastric mucosa; while sufficiently sensitive to detect orally administered, purified VacA even in rare myeloid cell populations, our highly specific and sensitive antiserum unfortunately did not allow tracking of H. pylori-derived VacA in the context of a live infection with either strain PMSS1 (which expresses s2m2 VacA) or strain J166 (which expresses s1m1 VacA). Purified VacA was taken up by gastric macrophages, as well as by two populations of DCs, i.e., CD103^+^ BATF-dependent DCs and CD11b^+^ IRF4-dependent DCs. All three populations are known to be recruited to the H. pylori-infected gastric mucosa (32). Gene expression analysis of CD11b^+^ DCs and macrophages—the numerically most abundant of the three myeloid populations—revealed clear differential responses to VacA-positive and –negative bacteria. In particular, we found that the level of expression of IL-23 by CD11b^+^ DCs is suppressed in a VacA-dependent manner, which correlates well with the higher frequencies of Th17 cells in the lamina propria of VacA mutant-infected relative to WT-infected mice. CD11b^+^ DCs are known to drive Th17 differentiation and/or maintenance (33), and IL-23 is a critical cytokine involved in the defense against intestinal bacterial pathogens such as Citrobacter rodentium, with CD11b^+^ DCs serving as a major source of IL-23 in the intestines (34). Our observation that VacA targets the CD11b^+^ DC/IL-23/Th17 axis to maintain high-level persistent infection is consistent with the results from the Citrobacter rodentium infection model and explains why a ΔVacA mutant is controlled more efficiently in mice. In macrophages, the dominant VacA-dependent effects on cytokine gene expression were observed for IL-10 and TGF-β, which were strongly induced only by VacA-expressing bacteria *in vitro* and *in vivo*. TGF-β in particular is well known to promote Treg differentiation in the periphery, where TGF-β signaling synergizes with microbial signals and retinoic acid to drive Foxp3 expression in naive T cells (35, 36). Activation of the TGF-β signaling pathway in naive T cells leads to the binding of SMAD2 and SMAD3 to a response element, termed conserved noncoding sequence 1 (CNS1), which is part of a *Foxp3* intronic enhancer and is known to regulate Foxp3 expression in pTregs (37). The combined data obtained from gene expression analyses of macrophages and DCs are consistent with the notion that VacA has evolved to create a tolerogenic setting, favoring pTreg induction and preventing effector T-cell (especially Th17) responses. In the absence of VacA, an IL-23/Th17 axis that is driven by CD11b^+^ DCs, and accompanied by reduced peripheral Treg differentiation, promotes infection control. Our data are in line with earlier studies that have shown—using both *in vitro* coculture and adoptive DC transfer models—that H. pylori exposure skews DC responses toward priming Treg over Th17 differentiation (38, 39). We now link this activity of H. pylori to VacA *in vitro* and *in vivo*. VacA is one of two H. pylori immunomodulators that target dendritic cells to promote immune tolerance. The other factor is a secreted enzyme, γ-glutamyl-transpeptidase (gGT), that drives the production of glutamate from glutamine (23). Glutamate then binds to glutamate receptors expressed by dendritic cells, and the downstream signaling pathway suppresses the expression of IL-6 and other proinflammatory cytokines by dendritic cells and boosts their ability to induce the expression of Foxp3, and of regulatory cytokines, in cocultured naive T cells (23). We have no indication that the two pathways of VacA- and gGT-driven immunomodulation are mutually dependent; rather, H. pylori appears to have evolved the two factors independently. gGTs are expressed also by other non-*pylori Helicobacter* species (40), whereas VacA expression is believed to be restricted to gastric *Helicobacter* strains, such as H. felis (41).

The second objective of this work was to identify a plausible mechanism underlying the differential responses of neonatal and adult mice to H. pylori. These differential responses translate into vastly different outcomes of the *Helicobacter*-host interaction (20) and are recapitulated in human carriers of H. pylori (10); therefore, reliable biomarkers that would predict one outcome (benign coexistence) or the other (chronic inflammation and the associated pathology) are urgently needed to guide rational treatment decisions. The most striking differences in neonatal and adult infected mice are observed in the T-cell compartment, with little if any T-cell and, especially, effector T-cell infiltration apparent in the gastric mucosa of neonatally infected mice. As a consequence, ratios of effector T cells to regulatory T cells differ radically as a consequence of the age at the time of first exposure. Strikingly, similar differences in the T-cell compartment are observed in the lungs, where Tregs are overrepresented as a consequence of neonatal but not adult infection. As shifts in the ratios of effector T cells to Treg cells were detected in the lung and stomach, but not in the examined lymphoid tissues (mesenteric lymph nodes and spleen, data not shown), we suspect that a special connection must exist between these two tissues. Indeed, only neonatally infected but not adult infected mice are protected against experimentally induced allergic airway inflammation upon ovalbumin or house dust mite allergen exposure (15–17), and an inverse association of H. pylori with allergic asthma is more evident in children or in young adults than in older individuals in epidemiological studies (11, 12). Our data add to a growing understanding of microbial effects on the so-called gut-lung axis, a concept that stipulates that changes in the host microbiota, especially of the GI tract, have effects on immunity in the lung (42). The idea of the existence of the gut-lung axis is, for example, supported by the finding that antibiotic-induced alterations in the gut microbiota in early life increase the risk of developing allergic airway disease (43, 44). Metabolites, especially short-chain fatty acids such as acetate and butyrate, derived from fermenting gut-resident microbes are known to affect the severity of allergic airway inflammation in mouse models (45). In addition to microbial metabolites, structural ligands of resident microbes, such as lipopolysaccharide (LPS) and peptidoglycan, especially when absorbed by or leaking into deeper tissues, are known to influence circulating lymphocytes and contribute to the regulation of systemic immune responses (42). H. pylori is unique in that it has evolved to avoid recognition by innate immune receptors; its LPS is modified to evade detection by Toll-like receptor 4 (TLR4) (46), and its flagellin cannot be recognized by TLR5 due to a mutation in the TLR5-binding domain (47). Rather, H. pylori expresses various immunomodulators, such as the VacA protein, γ-glutamyl-transpeptidase, NapA protein, and others, to actively interfere with either the maturation and function of antigen-presenting cells or directly with T cells (21, 23, 24, 48, 49), affecting immunity not only locally at the site of infection but also systemically and at distant sites.

## MATERIALS AND METHODS

### Mice.

C57BL/6 and CX_3_CR1^+/GFP^ mice were obtained from Janvier and the Jackson Laboratories and were bred and maintained under specific pathogen-free conditions in accredited animal facilities at the University of Zurich. All animal experimentation was reviewed and approved by the Zurich Cantonal Veterinary Office (licenses ZH24/2013, ZH170/2014, ZH224/2014, and ZH140/2017).

### H. pylori infection and VacA purification and treatment.

The H. pylori PMSS1 strain used in this study for *in vivo* experimentation is a clinical isolate from a patient with duodenal ulcer and the parental strain of mouse-derivative Sydney strain 1 (SS1) (20). Its VacA-deficient mutant has been described previously (23). H. pylori cultures were grown on horse blood agar plates and in liquid culture as described previously (20). Cultures were routinely assessed by light microscopy for contamination, morphology, and motility. Mice were infected orally on two consecutive days with 10^7^ CFU H. pylori PMSS1 at 7 days or 6 weeks of age and analyzed 6 weeks later unless specified otherwise. H. pylori VacA (WT or a Δ6-27 mutant protein) was purified from culture supernatants of H. pylori strain 60190 expressing streptavidin (Strep)-tagged s1m1 VacA via the use of a Strep Tactin resin as described previously (50). VacA was either orally or intraperitoneally administered at a dose of 20 μg/mouse.

### Leukocyte isolation.

For LP leukocyte isolation, gastrointestinal tissues were opened longitudinally, washed, and cut into pieces. The pieces were incubated in phosphate-buffered saline (PBS)–10% bovine serum albumin (BSA)–5 mM EDTA at 37°C to remove epithelial cells. Tissue was digested at 37°C for 30 min in a shaking incubator with 15 mM HEPES, 500 U/ml of type IV collagenase (Sigma-Aldrich), and 0.05 mg ml^−1^ DNase I in supplemented RPMI 1640 medium. Cells were then pushed through a cell strainer and layered onto a 40%/80% Percoll gradient and centrifuged, and the interface was washed in PBS–0.5% BSA. Peyer’s patch cell suspensions were prepared by pushing cells through a cell strainer using a syringe plunger. Lung cell suspensions were prepared by perfusion of the lung with PBS, followed by pushing the lung through a cell strainer.

### Flow cytometry and T-cell restimulation.

Cells were stained with a fixable viability dye and a combination of the following antibodies, including anti-mouse CD45 (clone 30-F11), CD11c (N418), major histocompatibility complex class II (MHCII) (M5/114.15.2), F4/80 (BM8), CD103 (M2E7), CD11b (M1/70), CD4 (RM4-5), neuropilin-1 (3E12), IL-17a (TC11-18H10.1), gamma interferon (IFN-γ) (XMG1.2), Ly6G (1A8), Ly6C (HK1.4), or an IgG isotype control (all from BioLegend), as well as TIM3 (FAB1529P; R&D). Fc block (anti-CD16/CD32; Affymetrix) was included to minimize nonspecific antibody binding. For intracellular cytokine staining, the cells were incubated at 37°C for 3 h in complete IMDM medium containing 0.1 μM phorbol 12-myristate 13-acetate (PMA) and 1 μM ionomycin with 1:1,000 brefeldin A (eBioscience) and GolgiStop solutions (BD Biosciences) at 37°C in a humidified incubator with 5% CO_2_. For the intranuclear staining of transcription factors, cells were fixed and permeabilized with a Foxp3/transcription factor staining buffer set (eBioscience) after surface staining was performed according to the manufacturer’s instructions. Cells were stained for 50 min with antibodies to FoxP3 (FJK-16s), RORγt (B2D) from Invitrogen, and Tbet (4B10) (from Biolegend). For VacA-specific staining, samples were fixed and permeabilized and stained with a rabbit antiserum specific for s1m1 VacA, which was described previously (28). Samples were acquired on a LSRII Fortessa flow cytometer (BD Biosciences) or sorted on an Aria III cell sorter (BD Biosciences) and analyzed using FlowJo software.

### *In vitro* macrophage cultures and infection.

Bone marrow cells were harvested from the femurs of multiple donor mice, seeded at 10^6^ cells/well in 1 ml of RPMI medium–10% FCS–penicillin (Pen)–streptavidin (Strep) and cultured with 20 ng/ml macrophage colony-stimulating factor (M-CSF) (PeproTech) for 6 days. Cells were washed and infected with H. pylori PMSS1 or H. pylori PMSS1 ΔVacA, G27, or G27ΔVacA at multiplicities of infection (MOIs) of 50 and 100 for 6 h or treated with 1 or 10 μg/ml purified VacA; supernatants were harvested and analyzed by IL-10 enzyme-linked immunosorbent assay (ELISA), and RNA from cells was isolated by the use of an RNeasy minikit (Qiagen) according to the manufacturer’s instructions.

### Quantitative PCR.

RNA was isolated from FACS-sorted cells using an RNeasy minikit (Qiagen) according to the manufacturer's instructions. cDNA synthesis was performed using SuperScript III reverse transcriptase (Qiagen). Quantitative PCRs for the candidate genes were performed using TaqMan gene expression assays as follows: for Hprt, Mm03024075_m1; for Tgfb1, Mm01178820_m1; for IL-10, Mm01288386_m1; for IL-23, Mm00518984_m1; for Il6, Mm00446190_m1; for Ptgs2, Mm00478374_m1; for Tnf, Mm00443258_m1; for IL-12b, Mm01288989_m1) (all from Applied Biosystems by Thermo Fisher Scientific). cDNA samples were analyzed in duplicate using a Light Cycler 480 detection system (Roche), and gene expression levels were normalized to hypoxanthine phosphoribosyltransferase (HPRT) expression for each sample. Mean relative gene expression levels were determined, and the differences were calculated using the threshold cycle (2Δ*C_T_*) method.

### Statistical analysis.

Statistical analysis was performed with Prism 6.0 (GraphPad Software). One-way analysis of variance (ANOVA) followed by pairwise comparisons and Dunn’s multiple comparison correction was used for all statistical comparisons of three or more groups. Differences were considered statistically significant with *P* values of <0.05 (*, *P* < 0.05; **, *P* < 0.01; ***, *P* < 0.005; ****, *P* < 0.001).
