# Supplementary figures and images for: Helicobacter pylori VacA Targets Myeloid Cells in the Gastric Lamina Propria To Promote Peripherally Induced Regulatory T-Cell Differentiation and Persistent Infection
Source: mBio. 2019 Mar 19;10(2):e00261-19. doi: 10.1128/mBio.00261-19 (PMC6426600; doi:10.1128/mBio.00261-19)

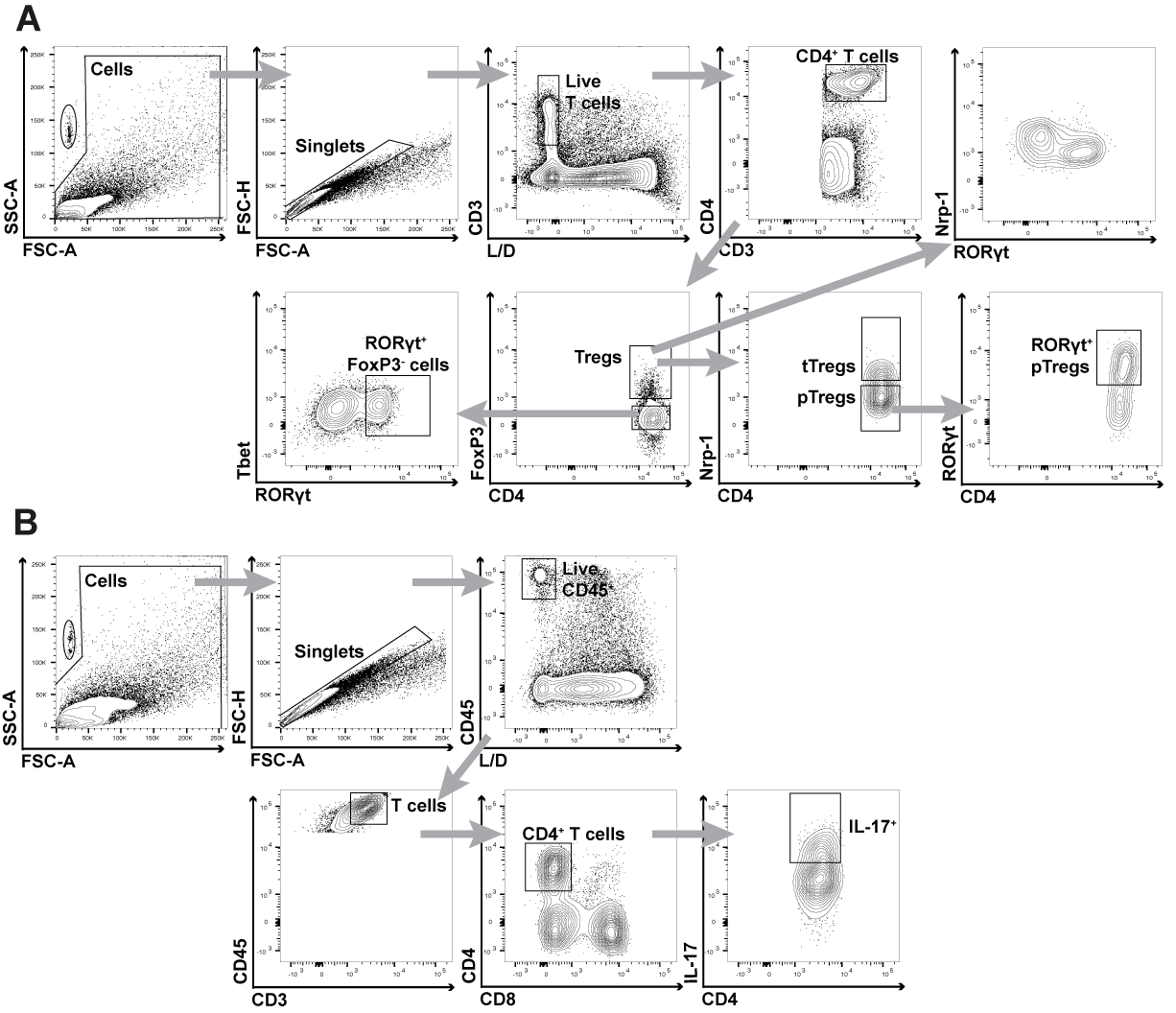

Supplement: FIG S1 [file mBio.00261-19-sf001.tif]

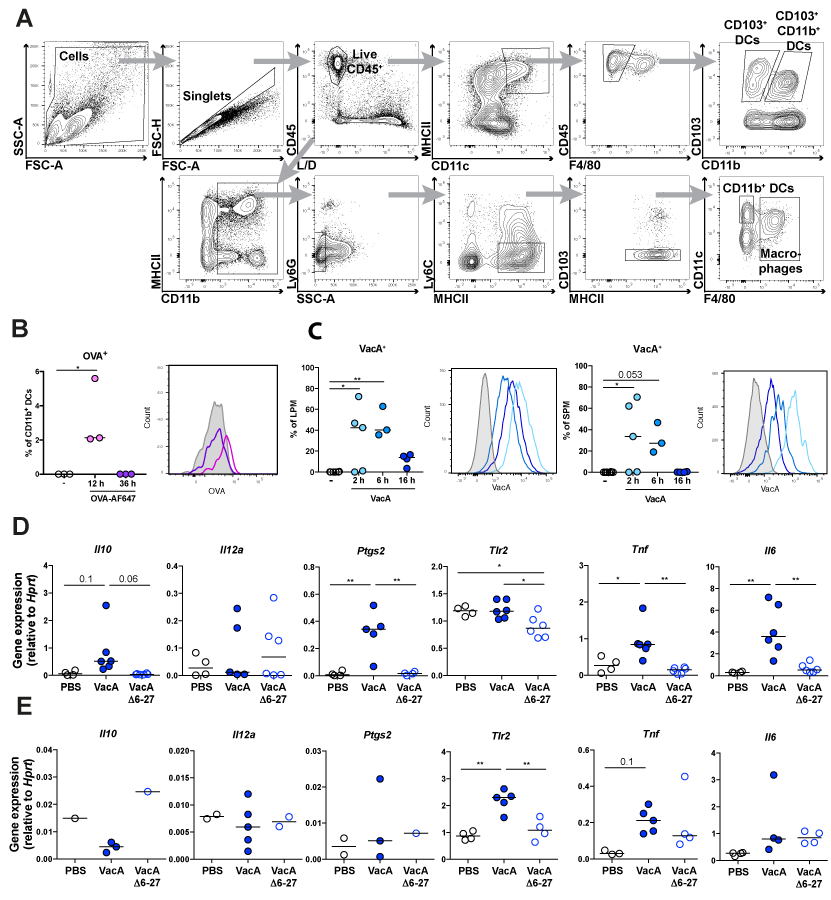

Supplement: FIG S2 [file mBio.00261-19-sf002.tif]

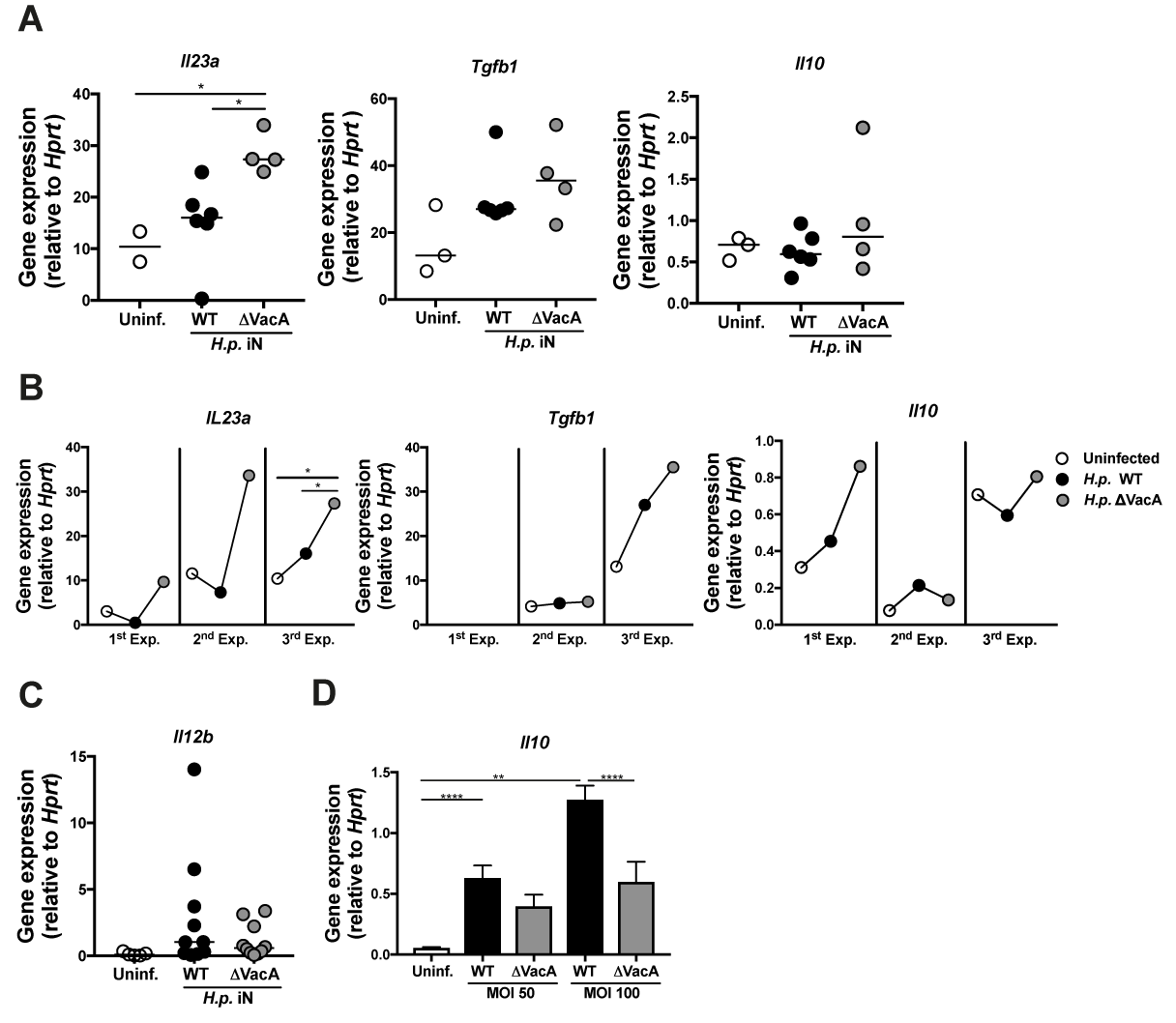

Supplement: FIG S3 [file mBio.00261-19-sf003.tif]

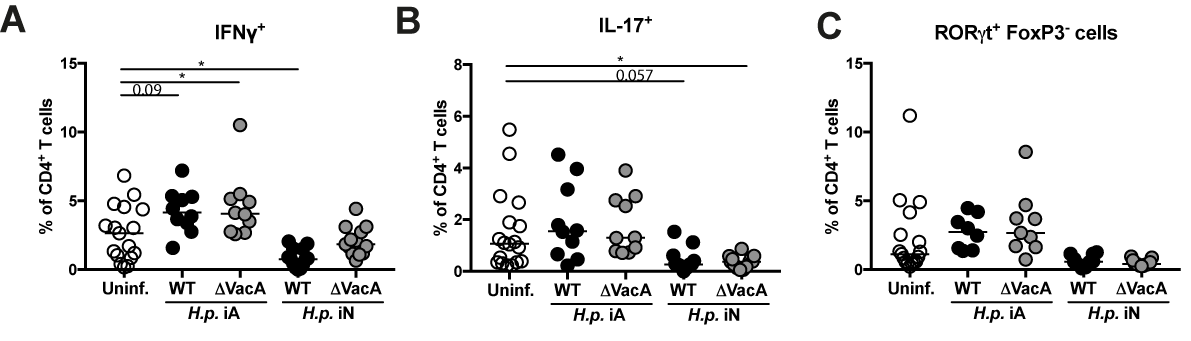

Supplement: FIG S4 [file mBio.00261-19-sf004.tif]
